# Bacterial Flora in Screw-Fixed Superstructures with Different Sealing Materials: A Comparative Clinical Trial

**DOI:** 10.3390/bioengineering11020195

**Published:** 2024-02-19

**Authors:** Kikue Yamaguchi, Motohiro Munakata, Kota Ishii, Takashi Uesugi

**Affiliations:** Department of Implant Dentistry, Showa University School of Dentistry, 2-1-1 Kita-senzoku, Ota-ku, Tokyo 1458515, Japan

**Keywords:** peri-implantitis, microleakage, screw access hole, glass ionomer cement, bacterial flora

## Abstract

A screw-fixed superstructure is predominantly selected for implant prostheses because of the concern regarding developing peri-implantitis, although its infection route remains unclear. Focusing on microleakage from access holes, the present study clinically investigated the bacterial flora in access holes with different sealing materials. We examined 38 sites in 19 patients with two adjacent screw-fixed superstructures. Composite resin was used in the control group, and zinc-containing glass ionomer cement was used in the test group. Bacteria were collected from the access holes 28 days after superstructure placement and were subjected to DNA hybridization analysis. The same patient comparisons of the bacterial counts showed a significant decrease in 14 bacterial species for the red, yellow, and purple complexes in the test group (*p* < 0.05). In addition, the same patient comparisons of the bacterial ratios showed a significant decrease in six bacterial species for the orange, green, yellow, and purple complexes in the test group (*p* < 0.05). Furthermore, the same patient comparisons of the implant positivity rates showed a significant decrease in the six bacterial species for the orange, yellow, and purple complexes in the test group. The results of this study indicate that zinc-containing glass ionomer cement is effective as a sealing material for access holes.

## 1. Introduction

Currently, dental implant treatment is selected for single, partial, and full jaw defect prostheses and is a highly predictive treatment with a high long-term survival rate. However, this high survival rate is influenced by various factors, including operator-dependent factors, patient-dependent factors, and implant-component factors [1,2,3]. With the recent increase in the number of implant-treatment patients, there has also been an increase in the incidence rate of peri-implant diseases, such as peri-implant mucositis and peri-implantitis, during the maintenance period [4,5].

Healthy peri-implant tissues are essential for the long-term maintenance and stability of implants. Peri-implant mucositis is the inflammation of the surrounding tissues without peri-implant bone loss. Peri-implantitis is an inflammation of the surrounding tissues accompanied by the loss of the peri-implant alveolar bone, which has been reported to be caused by bacterial infection in the oral cavity. Thus, screw-retained prostheses are currently selected for implant prostheses because they pose a lower risk of developing peri-implantitis caused by residual cement and offer easier management and recovery in cases of surrounding inflammation.

Regarding bacterial flora, some studies have reported differences between periodontitis and peri-implantitis, while others have reported otherwise; there have been a range of reports on the differences in the bacterial layers [6,7,8]. In particular, differences in periodontal-disease bacterial flora in the red and orange complexes have been indicated; however, it is unclear where this bacterial invasion occurs [9]. Furthermore, periodontal-disease bacterial flora in the red and purple complexes have been suggested to differ between healthy implants and peri-implantitis, although it remains unclear where individual bacteria invade.

Among the components of the dental implant system, the implant–abutment interface (IAI) and screw access hole (SAH) have been reported as sites of bacterial microleakage. However, previous studies have focused only on microleakage in the IAI [10,11], with few reports of bacterial invasion through access holes. Kofron et al. reported that the main disadvantage of the two-piece implant system lies in the presence of micro-gaps along the IAI, even though the abutment is fixed to the implant body by an abutment screw, and that a micro-gap, sized 10 and 135 µm, may cause biological and mechanical complications [4]. Considering the average size of bacteria (width: 0.2–1.5 μm, length: 1–10 μm) [12,13] and the aforementioned size of a micro-gap, it is clear that the space between an abutment and an implant function as a reservoir for bacteria. Consequently, bacteria are transported into and out of the implant body through the IAI, owing to the micro-movement of the abutment. As reported by Jervøe-Storm et al. on the contamination inside an implant following the removal of the abutment of a cement-retained prosthesis [14], infection of the peri-implant tissues can be attributed not only to bacterial invasion from the peri-implant groove but also to bacterial microleakage from the junction owing to the micro-gap and micro-movements of superstructures.

Regarding the bacterial flora with different SAH-sealing methods, do Nascimento et al. applied different temporary sealing methods for single, screw-fixed superstructures [15] and reported that a combination of polytetrafluoroethylene (PTFE) tape and light-polymerized resin resulted in the lowest mean bacterial count, whereas a combination of cotton pellet and instant polymerization resin resulted in the highest mean bacterial count. This indicates that the bacterial count inside an implant body varies greatly depending on the method used to seal the access hole. Furthermore, an in vitro study conducted by Park et al. showed that microleakage occurred only from access holes and not from the IAI [16], suggesting that microleakage from access holes is involved in the development of peri-implantitis. Therefore, the present study focused on the bacterial flora in access holes of screw-fixed superstructures and examined the differences in bacterial flora when two types of sealing materials were used to connect crowns in the same patients.

## 2. Materials and Methods

This study was approved by the Institutional Review Board of the Showa University Dental Hospital (approval no. DH2020-09; approval date, 28 July 2020). This study was conducted in accordance with the principles of the Declaration of Helsinki. All participants provided written informed consent to participate in this study. Informed consent was obtained from all participants involved in the study.

### 2.1. Participant Selection

All patients underwent placement surgery of two adjacent implants at the Department of Implant Dentistry at Showa University Dental Hospital, and those participating in the study were randomly selected from patients aged between 20 and 80 years who wore the final superstructures. A bone-level implant (Straumann) was used as the implant system for all patients, and connected zirconia superstructures of two adjacent teeth with screw-retained abutments were examined.

Exclusion criteria were as follows: Fully edentulous patients; patients with periodontal disease or diabetes mellitus; patients receiving radiation therapy or orthodontic treatment; patients who were pregnant or breastfeeding; patients who had undergone bone grafting at the time of implant placement; patients with bruxism; patients requiring prophylactic antibiotics or who were under steroid medication; patients who had marginal bone loss at the time of superstructure placement; patients wearing a prosthetic device without an intervening abutment (Table 1). These were modified with reference to the criteria of Zhang et al. [17].

#### 2.1.1. Superstructure

After the placement of the abutment, the titanium base and connecting crown (Zirconia, GeoMedi Co., Ltd., Fukuoka, Japan) were bonded using resin cement (3M, Saint Paul MN, USA) on the verification model. In addition, they were immersed in an H2O_2_ solution before being placed in the oral cavity.

#### 2.1.2. Placement of the Final Superstructures and Sealing Materials (Figure 1)

The twisted PTFE was pressure-welded to the lower part of the access hole of the final superstructure. For the upper part, (1) a dental composite resin (CR: SHOFU INC, Kyoto, Japan) (control group) or (2) zinc-containing glass ionomer cement (GI: GC Corporation, Tokyo, Japan; (test group) was placed in either the medial or distal access hole of the connected superstructures of the two adjacent teeth in a randomized manner and sealed.

**Figure 1:**
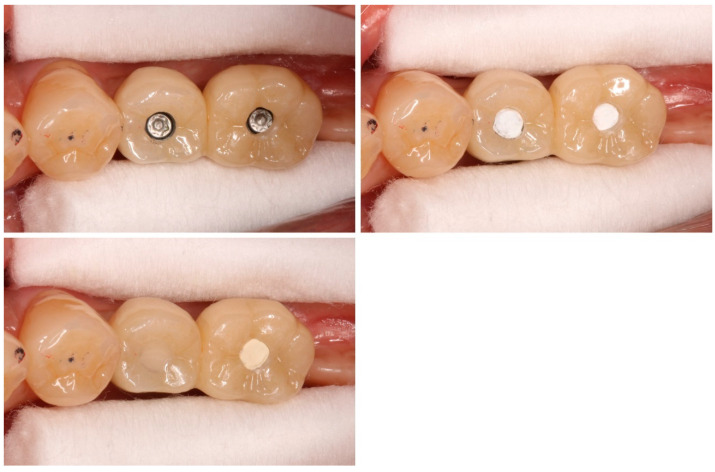
Final superstructures. The final superstructures are placed in the oral cavity, and the twisted PTFE is pressure-welded to the lower part of the access hole. The upper part is sealed after placing (**left**) CR (control group) or (**right**) GI (test group) in a randomized manner.

#### 2.1.3. Zinc-containing Glass Ionomer (GI) Cement

The GI cement released F, Zn, and Ca ions from the filling site. Zn ions suppress bacterial acid production [18], decalcification [19], and degradation of MMP-derived collagen [20]. F and Ca ions are known to suppress decalcification and promote recalcification; when combined with Zn ions, they are expected to exert multiple effects. In addition, an antibacterial test has shown that GI cement suppresses the growth of *S. mutans*, *S. sobrinus*, and other bacteria [21], and a biofilm formation test on material surfaces demonstrated that it exhibits effective anti-biofilm properties by reducing bacterial adhesion [22].

### 2.2. Sampling

The PTFE tape in the access holes was collected 28 days after placement of the superstructures and subjected to DNA hybridization analysis.

### 2.3. DNA Hybridization

As the first step of quantitative detection, we measured the total amount of 16S rRNA using the standard calibration curve plotted by Yazawa et al. [23]. Next, we determined the number of each bacterial species using a species-specific probe SI corrected with the hybridization affinity ratio.

Data from the Ribosomal RNA Database version 5.5 (Ann Arbor, MI, USA) were used to determine the number of copies of 16SrRNA. Without appropriate information, the median value for the genus was used. To calculate the total number of bacteria in the samples, 16S rRNA copy numbers relative to genomic DNA were assumed to be 4.5, which was calculated based on a weighted average reported in a study wherein the predominant and prevalent bacterial species in the saliva of orally healthy participants were determined using pyrosequencing [24]. The bacterial counts were calculated by multiplying Avogadro’s constant with the molecular weight of the genome (i.e., the molecular weight of 16S rRNA was divided by the number of 16S rRNA copies).

### 2.4. Items for Investigation

**Figure 2:**
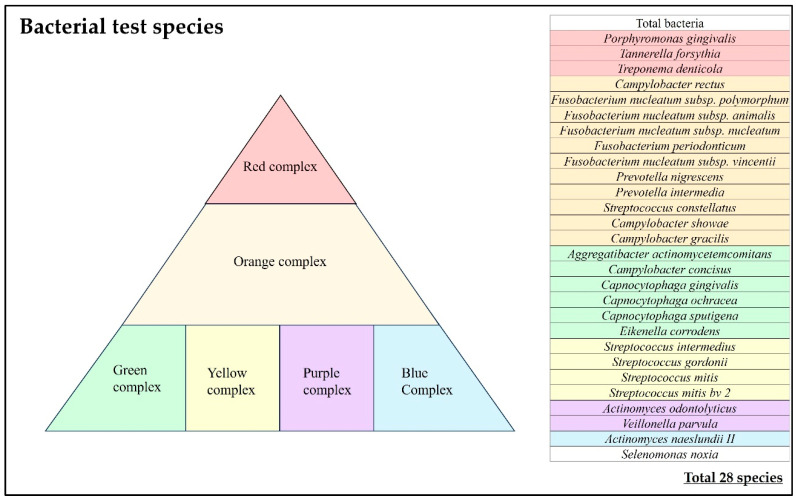
Bacteria test species.

For the 28 bacterial species and 34 items shown in Figure 2, we examined the (i) bacterial count, (ii) bacterial ratio, and (iii) implant positivity rate, which were compared between sealing materials and between the same individuals.

### 2.5. Date Analysis

Data were analyzed using statistical software (IBM SPSS Statics 29; IBM, Tokyo, Japan). The Wilcoxon signed-rank test was used to evaluate and compare the sealing materials, and the Mann–Whitney U test was used to evaluate and compare data between individuals. Statistical significance was set at *p* < 0.05.

## 3. Results

We examined superstructures (access holes, at 38 sites in 19 patients (Table 2).

### 3.1. Differences between Sealing Materials

#### 3.1.1. Bacterial Count (Table 3)

In the test group, a significant decrease in bacterial count was observed in total bacteria, one species of the red complex (*T. denticola* (*p* = 0.019 < 0.05)), total red complex, two species of the orange complex (*F. nucleatum* subsp. *vincentii* (*p* = 0.034, <0.05)*,* and *C. gracilis* (*p* = 0.044, <0.05)), total orange complex, one species of the green complex (*C. concisus* (*p* = 0.017, <0.05)), total green complex, one species of the yellow complex (*S. gordonii* (*p* = 0.0059, <0.01)), total yellow complex, one species of the purple complex (*V. parvula* (*p* = 0.029, <0.05)), and total purple complex. In the test group, 12 of 34 items and 6 out of 28 bacterial species showed a significant decrease in the bacterial count.

In particular, a marked decrease in the bacterial count was observed in the total bacteria, *S. gordonii,* and the total purple complex.

**Table 3:** Differences in bacteria count between sealing materials.

	Control	Test
Total Bacteria	13.0 × 10^6^	4.3 × 10^6^ **
*Porphyromonas gingivalis*	57,920	107,953
*Tannerella forsythia*	134,959	61,406
*Treponema denticola*	193,136	46,575 *
Red Complex	386,014	215,934 *
*Campylobacter rectus*	151,445	36,014
*Fusobacterium nucleatum* subsp. *polymorphum*	76,099	43,432
*Fusobacterium nucleatum* subsp. *animalis*	434,247	157,379
*Fusobacterium nucleatum* subsp. *nucleatum*	112,125	88,747
*Fusobacterium periodonticum*	114,794	48,227
*Fusobacterium nucleatum* subsp. *vincentii*	42,976	18,606 *
*Prevotella nigrescens*	110,071	40,947
*Prevotella intermedia*	70,909	123,354
*Streptococcus constellatus*	25,113	37,062
*Campylobacter showae*	17,134	3481
*Campylobacter gracilis*	40,177	3371 *
Orange Complex	1,195,088	600,620 *
*Aggregatibacter actinomycetemcomitans*	462	66
*Campylobacter concisus*	28,056	3503 *
*Capnocytophaga gingivalis*	5081	1624
*Capnocytophaga ochracea*	28,994	1410
*Capnocytophaga sputigena*	54,692	6540
*Eikenella corrodens*	3004	4051
Green Complex	120,290	17,194 *
*Streptococcus intermedius*	6942	189
*Streptococcus gordonii*	104,047	17,282 **
*Streptococcus mitis*	22,251	23,349
*Streptococcus mitis bv 2*	19,752	20,367
Yellow Complex	152,992	61,186 *
*Actinomyces odontolyticus*	1707	1529
*Veillonella parvula*	110,860	46,849 *
Purple Complex	112,567	48,377 **
*Actinomyces naeslundii II*	77,696	44,997
*Selenomonas noxia*	22,013	2144

* *p* < 0.05, ** *p* < 0.01.

#### 3.1.2. Bacterial Ratio (Table 4)

In the test group, a significant decrease in the bacterial ratio was observed in *C. gracilis* (*p* = 0.05) of the orange complex, *C. concisus* (*p* = 0.00, <0.05) of the green complex, and the total green complex. In the test group, 3 of 32 items and 2 of 28 bacterial species showed a significant decrease in the bacterial ratio.

**Table 4:** Differences in bacterial ratio between sealing materials.

	Control	Test
Total Bacteria		
*Porphyromonas gingivalis*	0.24	0.23
*Tannerella forsythia*	0.50	0.00
*Treponema denticola*	0.64	0.00
Red Complex	4.36	0.23
*Campylobacter rectus*	0.48	0.00
*Fusobacterium nucleatum* subsp. *polymorphum*	0.38	0.19
*Fusobacterium nucleatum* subsp. *animalis*	1.46	0.00
*Fusobacterium nucleatum* subsp. *nucleatum*	0.35	0.00
*Fusobacterium periodonticum*	0.49	0.27
*Fusobacterium nucleatum* subsp. *vincentii*	0.18	0.01
*Prevotella nigrescens*	0.40	0.02
*Prevotella intermedia*	0.18	0.00
*Streptococcus constellatus*	0.11	0.00
*Campylobacter showae*	0.08	0.00
*Campylobacter gracilis*	0.24	0.05 *
Orange Complex	4.36	0.54
*Aggregatibacter actinomycetemcomitans*	0.00	0.00
*Campylobacter concisus*	0.24	0.00 *
*Capnocytophaga gingivalis*	0.03	0.01
*Capnocytophaga ochracea*	0.18	0.00
*Capnocytophaga sputigena*	0.25	0.43
*Eikenella corrodens*	0.02	0.00
Green Complex	0.72	0.44 *
*Streptococcus intermedius*	0.05	1.51
*Streptococcus gordonii*	1.01	0.55
*Streptococcus mitis*	0.20	0.00
*Streptococcus mitis bv 2*	0.19	1.34 *
Yellow Complex	1.45	3.40
*Actinomyces odontolyticus*	0.02	2.91 *
*Veillonella parvula*	2.32	0.16
Purple Complex	2.34	3.07
*Actinomyces naeslundii II*	0.93	2.31
*Selenomonas noxia*	0.09	1.00

* *p* < 0.05.

#### 3.1.3. Implant Positivity Rate (Table 5)

In the test group, a significant decrease in implant positivity rate was observed in four species of the orange complex (*F. nucleatum* subsp. *animalis*, *F. periodonticum, F. nucleatum* subsp. *vincentii*, and *C. gracilis*), total orange complex, one species of the green complex (*C. concisus*), and total green complex.

In the test group, 7 of 34 items and 5 of 28 bacterial species showed a significant decrease in the implant positivity rate. In particular, a marked decrease in the implant positivity rate was observed for the total orange and green complexes.

**Table 5:** Differences in implant positivity rate between sealing materials.

	Control	Test
Total Bacteria		
*Porphyromonas gingivalis*	26.3%	15.8%
*Tannerella forsythia*	78.9%	78.9%
*Treponema denticola*	84.2%	63.2%
Red Complex	63.2%	52.6%
*Campylobacter rectus*	26.3%	26.3%
*Fusobacterium nucleatum* subsp. *polymorphum*	42.1%	15.8%
*Fusobacterium nucleatum* subsp. *animalis*	47.4%	15.8% *
*Fusobacterium nucleatum* subsp. *nucleatum*	57.9%	31.6%
*Fusobacterium periodonticum*	47.4%	15.8% *
*Fusobacterium nucleatum* subsp. *vincentii*	52.6%	15.8% *
*Prevotella nigrescens*	31.6%	21.1%
*Prevotella intermedia*	26.3%	15.8%
*Streptococcus constellatus*	42.1%	36.8%
*Campylobacter showae*	31.6%	10.5%
*Campylobacter gracilis*	42.1%	10.5% *
Orange Complex	40.7%	19.6% **
*Aggregatibacter actinomycetemcomitans*	10.5%	5.3%
*Campylobacter concisus*	57.9%	21.1% *
*Capnocytophaga gingivalis*	36.8%	15.8%
*Capnocytophaga ochracea*	31.6%	10.5%
*Capnocytophaga sputigena*	36.8%	21.1%
*Eikenella corrodens*	21.1%	10.5%
Green Complex	32.5%	14.0% **
*Streptococcus intermedius*	42.1%	15.8%
*Streptococcus gordonii*	89.5%	78.9%
*Streptococcus mitis*	89.5%	84.2%
*Streptococcus mitis bv 2*	94.7%	89.5%
Yellow Complex	78.9%	67.1% *
*Actinomyces odontolyticus*	89.5%	89.5%
*Veillonella parvula*	73.7%	47.4% *
Purple Complex	81.6%	68.4%
*Actinomyces naeslundii II*	89.5%	89.5%
*Selenomonas noxia*	36.8%	15.8%

* *p* < 0.05, ** *p* < 0.01.

### 3.2. Differences between Patients

#### 3.2.1. Bacterial Count (Table 6)

In the test group, a significant decrease in bacterial count was observed in total bacteria, one species of the red complex (*T. denticola*), total red complex, eight species of the orange complex (*F. nucleatum* subsp. *polymorphum*, *F. nucleatum* subsp. *animals*, *F. nucleatum* subsp. *nucleatum*, *F. periodonticum*, *F. nucleatum* subsp. *vincentii*, *P. nigrescens*, *C. showae*, and *C. gracilis*), total orange complex, two species of the green complex (*C. concisus* and *Capnocytophaga ochracea*), total green complex, two species of the yellow complex (*S. intermedius* and *S. gordonii*), total yellow complex, two species of the purple complex (*A. odontolyticus* and *V. parvula*), total purple complex, and two species of the blue complex (*A. naeslundii II* and *Selenomonas noxia*).

In the same patient comparisons, the test group showed a significant decrease in bacterial counts in 23 out of 34 items and 17 out of 28 bacterial species.

**Table 6:** Differences in the same individuals.

	Bacterial Count	Bacterial Ratio
Total Bacteria	0.000091 **	
*Porphyromonas gingivalis*	0.68	0.344
*Tannerella forsythia*	0.068	0.159
*Treponema denticola*	0.003 **	0.398
Red Complex	0.029 *	0.621
*Campylobacter rectus*	0.368	0.368
*Fusobacterium nucleatum* subsp. *polymorphum*	0.0058 **	0.288
*Fusobacterium nucleatum* subsp. *animalis*	0.011 *	0.043 *
*Fusobacterium nucleatum* subsp. *nucleatum*	0.025 *	0.628
*Fusobacterium periodonticum*	0.0038 **	0.26
*Fusobacterium nucleatum* subsp. *vincentii*	0.0026 **	0.194
*Prevotella nigrescens*	0.046 *	0.252
*Prevotella intermedia*	0.681	0.344
*Streptococcus constellatus*	0.297	0.054
*Campylobacter showae*	0.014 *	0.014 *
*Campylobacter gracilis*	0.0058 **	0.0086 **
Orange Complex	0.025 *	0.943
*Aggregatibacter actinomycetemcomitans*	0.328	0.328
*Campylobacter concisus*	0.017 *	0.123
*Capnocytophaga gingivalis*	0.104	0.338
*Capnocytophaga ochracea*	0.015 *	0.014 *
*Capnocytophaga sputigena*	0.034	0.2
*Eikenella corrodens*	0.68	0.344
Green Complex	0.018 *	0.127
*Streptococcus intermedius*	0.0086 **	0.288
*Streptococcus gordonii*	0.0005 **	0.128
*Streptococcus mitis*	0.293	0.0049 **
*Streptococcus mitis bv 2*	0.357	0.0033 **
Yellow Complex	0.0069 **	0.188
*Actinomyces odontolyticus*	0.043 *	0.0078 **
*Veillonella parvula*	0.00061 **	0.018 *
Purple Complex	0.000091 **	0.063
*Actinomyces naeslundii II*	0.043 **	0.099
*Selenomonas noxia*	0.0086 **	0.018 *

* *p* < 0.05, ** *p* < 0.01.

#### 3.2.2. Bacterial Ratio (Table 6)

In the test group, a significant decrease in bacterial ratio was observed in three species of the orange complex (*F. nucleatum* subsp. *animalis*, *C. showae*, and *C. gracilis*), two species of the yellow complex (*Capnocytophaga ochracea* and *S. mitis*), one species of the green complex (*Capnocytophaga ochracea*), one species of the purple complex (*Veillonella parvula*), and *Selenomonas noxia*.

In the same patient comparisons, the test group showed a significant decrease in the bacterial ratio in 8 of 28 bacterial species.

#### 3.2.3. Implant Positivity Rate (Figure 3)

The implant positivity rate was 51.3% in the control group and 34.2% in the test group, thereby showing a significant decrease in the test group (*p* = 0.0019, <0.005).

**Figure 3:**
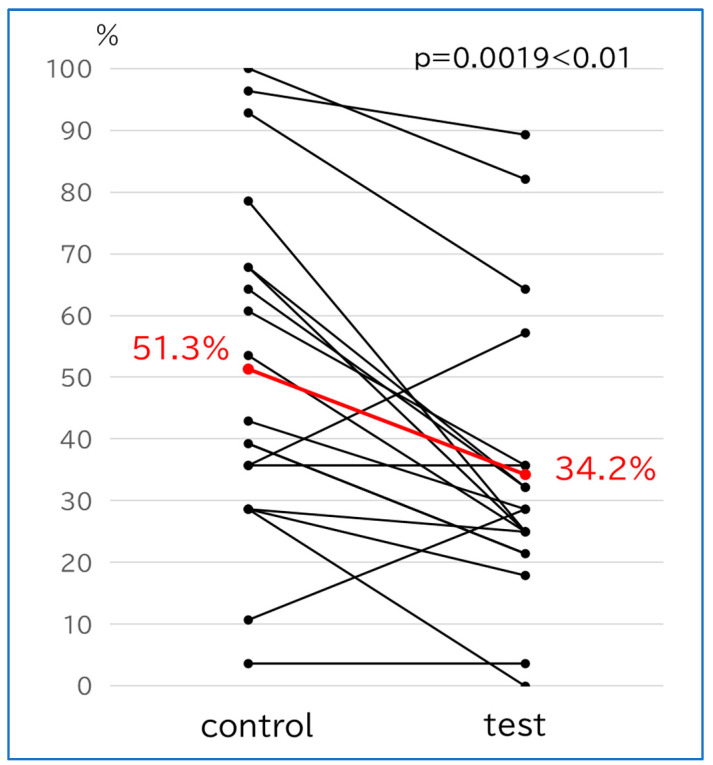
Differences in implant positivity rate in the same individual patients. The red lines indicate the respective averages.

## 4. Discussion

In bacteriological research on peri-implantitis, oral bacteria of the “red” and “orange” complexes are believed to be closely associated with periodontal disease and peri-implantitis. Furthermore, there have been many recent reports on the differences in bacterial flora between periodontitis and peri-implantitis and between healthy implants and peri-implantitis [6,7,8]. In addition, a review published in 2021 stated that the bacterial flora for peri-implantitis differs from that of periodontitis and that a comparison of peri-implant health showed a similar trend in the involvement of many types of bacteria and differences in bacterial species [9]. For example, the bacterial flora common to periodontitis and peri-implantitis includes *P. gingivalis* in the red complex, *Fusobacterium* spp. in the orange complex, and *Streptococcus* spp. in the yellow complex, whereas bacterial flora unique to peri-implantitis include *T. denticola* in the red complex and *P. nigrescens* in the orange complex. In addition, the bacterial floras common to healthy implants and peri-implantitis include *Fusobacterium* spp., *Campylobacter gracilis*, and *Streptococcus* spp. of the orange complex, while the bacterial flora unique to peri-implantitis include *T. denticola*, *P. gingivalis*, and *T. forsythia* of the red complex and *Actinomyces* of the purple complex.

Regarding the bacterial positivity rate, Cortelli et al. reported that peri-implantitis had a higher implant positivity rate for *T. forsythia*, *T. denticola*, and *P. intermedia* than periodontitis [25]. In contrast, Zhuang et al. reported no obvious difference in the implant positivity rates for *T. denticola* and *P. intermedia* [17]. Canullo et al. reported that compared to healthy implants, the bacterial flora in the internal connection of the implant after crown/abutment removal in peri-implantitis showed a greatly different implant positivity rate for *T. denticola* of the red complex, *P. intermedia* of the orange complex, and *E. corrodens* and *C. albicans* of the green complex [26].

There have been various reports on the bacterial flora in peri-implantitis, with different types of bacteria examined and bacterial analysis methods used (DNA probe analysis and PCR method). A review by Pérez-Chaparro et al. showed evidence for the association of peri-implantitis with *P. gingivalis*, *T. forsythia*, and *T. denticola* of the red complex, as well as the association of peri-implantitis with *P. intermedia* of the orange complex [27]. Furthermore, a systematic review by Lafaurie et al. reported that the red complex bacteria were detected at a slightly high frequency in the bacterial flora of peri-implantitis, that the orange complex bacteria, such as *P. intermedia* and *P. nigrescens*, were more commonly associated with peri-implantitis, that there was a low association of the red complex bacteria, and that uncultivable anaerobic Gram-positive bacteria, Gram-negative bacteria, and oral resident bacteria, such as *Staphylococcus aureus*, were also identified [28]. Since the present prospective study was conducted only on healthy implants, we could not examine the suppressive effect on the surrounding inflammation. However, sealing with glass ionomer cement, used in this study, was suggested to be highly effective in preventing peri-implantitis, as it suppressed subgingival bacterial flora, including the total red and orange complexes.

Regarding bacterial invasion from access holes into the implant body, the bone-level implant (two-piece implant system) used in this study had a superstructure and an abutment mechanically connected at or below the bone margin, thereby containing multiple routes for bacterial invasion, such as IAI and SAH. Moreover, sealing materials used in the access hole of the screw-retain superstructure are prone to intraoral contamination. Although various materials have been studied as sealing materials for SAH in screw-retained superstructures [16,29,30,31,32,33], few studies have focused on the capacity of materials to prevent or minimize microbial/bacterial leakage from SAH. Cavalcanti et al. compared gutta-percha (GP) and PTFE as sealing materials for the lower part of access holes, reporting that GP was significantly more effective than PTFE [32]. In contrast, Alshehri et al. reported that PTFE was significantly more effective than GP [33]. Furthermore, the insertion and removal of PTFE was clinically easy, although twisted PTFE had no sealing effect, even when compressed, owing to the lack of chemical bonds. Although GP can be easily compressed and chemically bonded, their insertion is difficult, and their removal is time-consuming. Thus, there are advantages and disadvantages to sealing materials for the lower part of access holes. The results of this study suggest that F, Zn, and Ca ions released from the site filled with GI cement may have acted in an anti-bacterial manner. In addition, we believe that GI cement achieved superior bacterial suppression compared to CR in this study because it can even be applied to sites where moisture-proofing is difficult, it does not require a bonding material, and it exhibits no polymerization shrinkage. Furthermore, our results suggest that reducing the bacterial count and implant positivity rate would suppress the development of peri-implantitis. In addition, future studies will examine differences in bacterial flora according to age.

## 5. Limitations

This study made comparisons not only between sealing materials but also between the same individuals, as each patient has different oral bacterial flora. However, because this study was conducted on healthy implants, we did not evaluate the association with the bacterial flora of peri-implantitis, the difference from the bacterial flora of the peri-implant gingival groove, and the difference in bacterial flora in patients with cement-retained prosthesis. In addition, because a superstructure is placed in the oral cavity for a long period of time, it is necessary to conduct long-term observational studies, including examination of attrition and abrasion, and further studies are needed in the future.

## 6. Conclusions

We compared the sealing materials for the upper part of the access holes of screw-retained superstructures. Our results showed that GI cement reduced the bacterial count in access holes and suppressed the implant positivity rate when compared with other materials and in the same patients. These results suggest that GI cement is a useful sealing material for access holes.

## Figures and Tables

**Table 1:** Inclusion and exclusion criteria.

Inclusion Criteria
Implants placed and superstructures was inserted at this hospital
Age: between 20 and 80 years
Written consent to participate in the research was obtained from the individual.
Two implants are adjacent to each other.
Connected superstructure inserted.
Exclusion criteria
Fully edentulous patients
Bone grafting was performed at the time of implant placement
Periodontal disease or diabetes mellitus
Undergoing or previously undergoing radiation therapy to the head and neck
Bruxism
Pregnancy, possible pregnancy, breastfeeding, or considering pregnancy
Patients requiring prophylactic antibiotics or who were taking steroid medications
Marginal bone loss at the time of superstructure attachment
Prosthetic devices without intervening abutments were inserted

**Table 2:** Patient data.

Gender	
Male	13
Female	6
Age (y)	
40–49	2
50–59	3
60–69	7
70–79	7
Mean ± SD	65.7 ± 10.87

## Data Availability

The datasets used and analyzed during the current study are available from the corresponding author upon reasonable request.
